# Implantable Cardioverter-defibrillator Magnetic Interference by the iPhone 12: Is It Clinically Significant?

**DOI:** 10.19102/icrm.2022.130702

**Published:** 2022-07-15

**Authors:** Harshil Patel, Cameron Whitler, Nathan Foster, Christopher Bradley, Dipak Shah, Christian Machado

**Affiliations:** ^1^Division of Cardiology, Ascension Providence Hospital/MSUCHM, Southfield, MI, USA

**Keywords:** ICD, magnetic interference, sudden cardiac arrest

## Abstract

The iPhone 12 series (Apple, Inc., Cupertino, CA, USA) contains a circular array of magnets around a central charging coil (compatible with “Magsafe” technology). The device was recently reported to have magnetic interference with implantable cardioverter-defibrillators (ICDs). We sought to test the electromagnetic interference of the iPhone 12 in inhibiting life-saving therapies of ICDs in clinical settings. After obtaining written informed consent, an iPhone 12 was placed over the device generators of 17 patients in the ICD clinic. Device interrogation was performed immediately before and after placing the iPhone over the ICD generator to evaluate for any inhibition of device therapies. To emulate a real-world scenario, the iPhone 12 was not placed directly over the skin above the device generator but instead was positioned over the patients’ clothes. None of the device interrogations revealed interruption of device therapies due to the iPhone. We concluded that, despite the iPhone having shown in vitro interference of ICD functioning, its effects are not clinically relevant in vivo. Larger studies need to be performed to confirm this finding and guide safety recommendations regarding the use of iPhones containing magnets by patients with implanted ICDs.

## Introduction

The iPhone 12 series (Apple, Inc., Cupertino, CA, USA), containing a circular array of magnets around a central charging coil (compatible with the “MagSafe” technology), was recently reported to cause magnetic interference with an implantable cardioverter-defibrillator (ICD).^1^ The MagSafe technology, which enables wireless charging functionality, consists of an array of magnets capable of generating a magnetic field of around 0.005 tesla.^2^ Studies that have reported interference with implantable devices by the iPhone 12 were not performed on ambulatory patients in a clinical setting. We present a case series of 17 patients with ICDs in whom testing of their device for interference from an iPhone 12 was carried out in an ICD clinic.

## Methods

This prospective study included 17 patients, all of whom were randomly selected. The study was conducted at the hospital device clinic after obtaining ethics committee approval. Written informed consent was obtained, after which each patient underwent a baseline device interrogation. An iPhone 12 in working condition with a fully charged battery, active WiFi, and active cellular data/calling services was used for conducting the study. The iPhone 12, without any phone cover, was placed over the ICD pocket above the clothing. The iPhone was held in 3 separate positions: directly above the device pocket and superior and inferior to the pocket, for 20 s in each position. Device interrogation was repeated to determine any change in settings or functionality.

## Results

None of the 17 patients had any demonstrable change in the settings or magnet response events upon repeat interrogation **(Table 1)**.

## Discussion

The magnet response in ICDs is the inhibition of tachyarrhythmia therapies, whereas the magnet response is asynchronous pacing in pacemakers.^3^

The MagSafe technology by Apple enables wireless charging capabilities of the iPhone. The charging coil is surrounded by an array of magnets. When the iPhone is placed on a charging pad with an inbuilt magnet, it is aligned with relative precision to allow wireless charging.

Recent studies by Greenberg et al. and Nadeem et al. demonstrated inhibition of lifesaving therapies by the Apple iPhone 12 Pro Max.^1,2^ After these reports were published, the iPhone manufacturer (Apple) released a statement detailing precautionary measures for iPhone users with a cardiac implantable electronic device (CIED) in place. Recommendations were also made to keep the iPhone 12 at a distance of ≥6 in (15 cm) from the CIED as a safety measure to prevent inhibition of lifesaving therapies.^4^ While the iPhone 12 Pro Max interfered with the signals when placed directly over the skin with a CIED generator underneath, our study could not replicate these findings when the phone was placed over clothing (rather than on the skin). The rationale of placing the device over the clothing was to mimic a more practical scenario, such as placing the phone in a shirt pocket.

The results of a recent study by Held et al. are similar to those of ours.^5^ In their case series of 12 patients, 4 different phones as well as an iPhone 12 case with an embedded magnet were used to evaluate the interference of implanted device signals. None of the 12 devices showed any interference in their signals.

These results raise pertinent questions regarding the clinical significance of recommendations of keeping the phone 6 in away from the device generator. Studies with measurable clinical outcomes like mortality from cardiac arrest due to the inhibition of tachycardia therapies would certainly help answer these questions. However, an ethical dilemma might arise when designing and implementing such a protocol. This would particularly be the case when the risk of inhibition of device therapies can be avoided by keeping the phone away from the device. The fact that studies have shown conflicting results on the risk of phones with magnets interfering with CIEDs’ functionality suggests that more data are certainly needed with multiple variables.^1,2,5^ The precise distance of the phone from the device, the thickness of the clothing, and the thickness of subcutaneous tissue over the generator are some of the factors that could have a significant impact on the conflicting results.

## Figures and Tables

**Table 1:** Device and Demographic Information of Study Participants

	Device Information			Patient Demographics			
Patient #	ICD Manufacturer	Device Model	Description	BMI (kg/m^2^)	Sex	Age (Years)	Pacemaker Dependence?
1	Boston Scientific (Natick, MA, USA)	Energen ICD E140	Single chamber	38.3	Female	61	No
2	Boston Scientific	Teligen 100 E110	Dual chamber	39.2	Male	50	No
3	Boston Scientific	Energen VR E141	Single chamber	25	Male	64	No
4	St. Jude Medical (St. Paul, MN, USA)	Ellipse VR 1411-36Q	Single chamber	44.1	Male	53	No
5	St. Jude Medical	Unify Assura 3357-40Q	Biventricular	23.3	Female	82	No
6	St. Jude Medical	Unify Assura 3357-40Q	Biventricular	22.9	Male	36	No
7	St. Jude Medical	Quadra Assura 3365-40Q	Biventricular	28	Female	73	No
8	Medtronic (Minneapolis, MN, USA)	Visia AF MRI VR DVFB1D4	Single chamber	26	Male	67	No
9	St. Jude Medical	Ellipse VR 1411-36Q	Single chamber	24	Male	62	No
10	Medtronic	Claria MRI CRT-D DTMA1Q1	Biventricular	28	Male	78	No
11	St. Jude Medical	Ellipse VR 1411-36	Single chamber	27	Female	84	No
12	St. Jude Medical	2411-36Q	Dual chamber	28	Male	80	No
13	Boston Scientific	Dynagen D150	Single chamber	35	Male	47	No
14	St. Jude Medical	Unify Assura 3357-40C	Biventricular	28	Female	49	No
15	St. Jude Medical	Ellipse VR 1411-36C	Single chamber	35	Male	76	No
16	St. Jude Medical	Ellipse VR 1411-36	Single chamber	22	Male	83	No
17	Medtronic	Claria MRI CRT-D DTMA1Q1	Biventricular	32	Female	55	No

*Abbreviations:* BMI, body mass index; ICD, implantable cardioverter-defibrillator.
